# The use of SMALPs as a novel membrane protein scaffold for structure study by negative stain electron microscopy

**DOI:** 10.1016/j.bbamem.2014.10.018

**Published:** 2015-02

**Authors:** Vincent Postis, Shaun Rawson, Jennifer K. Mitchell, Sarah C. Lee, Rosemary A. Parslow, Tim R. Dafforn, Stephen A. Baldwin, Stephen P. Muench

**Affiliations:** a School of Biomedical Sciences, Faculty of Biological Sciences, University of Leeds, LS2 9JT, UK; b Biomedicine Research Group, Faculty of Health and Social Sciences, Leeds Beckett University, LS1 3HE, UK; c School of Biosciences, University of Birmingham, Birmingham, Edgbaston B15 2TT, UK

**Keywords:** SMALP, Electron microscopy, Membrane protein, AcrB

## Abstract

Despite the great progress recently made in resolving their structures, investigation of the structural biology of membrane proteins still presents major challenges. Even with new technical advances such as lipidic cubic phase crystallisation, obtaining well-ordered crystals remains a significant hurdle in membrane protein X-ray crystallographic studies. As an alternative, electron microscopy has been shown to be capable of resolving > 3.5 Å resolution detail in membrane proteins of modest (~ 300 kDa) size, without the need for crystals. However, the conventional use of detergents for either approach presents several issues, including the possible effects on structure of removing the proteins from their natural membrane environment. As an alternative, it has recently been demonstrated that membrane proteins can be effectively isolated, in the absence of detergents, using a styrene maleic acid co-polymer (SMA). This approach yields SMA lipid particles (SMALPs) in which the membrane proteins are surrounded by a small disk of lipid bilayer encircled by polymer. Here we use the *Escherichia coli* secondary transporter AcrB as a model membrane protein to demonstrate how a SMALP scaffold can be used to visualise membrane proteins, embedded in a near-native lipid environment, by negative stain electron microscopy, yielding structures at a modest resolution in a short (days) timeframe. Moreover, we show that AcrB within a SMALP scaffold is significantly more active than the equivalent DDM stabilised form. The advantages of SMALP scaffolds within electron microscopy are discussed and we conclude that they may prove to be an important tool in studying membrane protein structure and function.

## Highlights

• Maintaining membrane proteins in a native-like environment is difficult.
• SMALP scaffolds efficiently extract AcrB from the membrane.
• We show SMALP scaffolds to be a robust tool for rapid structural analysis by EM.

## Graphical abstract

**Figure:**
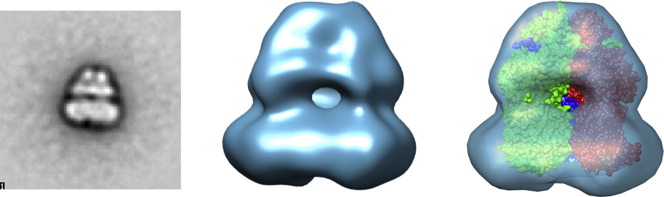

## 1 Introduction

Despite their physiological and pharmaceutical importance, membrane proteins still present a significant challenge in structural biology. They represent a major target in drug design because of their roles in transport across membranes and in signal transduction [1,2]. The primary reason for their challenging nature is the need to extract them from their natural environment, the lipid bilayer, and subsequently maintain them in stable, water-soluble form. Membrane proteins are typically purified and studied by using detergents, the amphipathic natures of which allow them to mimic the membrane environment [3]. Identifying optimal detergent(s) and buffer conditions for protein stability is, however, often difficult and time consuming [4,5]. In addition, this approach has a number of significant problems. Firstly, the surfactant micelle created by the detergent provides only a rough approximation of the natural membrane environment. Secondly, the growing information on the structure/function relationships of membrane proteins indicates that the lipid bilayer often does not simply hold the protein in place but can also intimately interact with the protein, modifying its function [6,7,8]. For example, changes in the membrane phospholipid composition, acyl chain saturation or drug binding to the lipids can have significant effects on the embedded proteins [9]. It follows that although the use of detergents facilitates the isolation of membrane proteins, the associated loss of the natural lipid environment can have profound effects on their structures and/or function.

The finding that the membrane environment around the protein is not simply a scaffold but can influence protein folding and function has triggered the development of alternative approaches for extraction and characterisation of membrane proteins. For example, amphipols use a strongly hydrophilic backbone, combined with hydrophobic side chains, to encapsulate a membrane protein in a hydrophobic environment whilst keeping a hydrophilic exterior [10]. Although they have been shown to stabilise many proteins, including bacteriorhodopsin, cytochrome b6f, GPCRs and Ca^2 +^ ATPase in an aqueous environment [10,11,12,], polymer aggregation problems and interference of the amphipol scaffold with protein function have been reported [11]. Alternatively, bicelles have been used to stabilise membrane proteins. Their bilayer nature, formed by the mixture of an amphiphile and long chain phospholipids in an aqueous environment, allows for a more native-like membrane environment, which can be amenable to crystallisation experiments [13,14]. It is important to note, however, that prior to transferring the protein into the bicelle, detergents are first required for extraction of the protein from the membrane. Hence this method is still hindered by the need to remove proteins, albeit briefly, from their natural membrane environment. The same problem is encountered in preparation of membrane-mimetic nanodiscs. These consist of amphipathic helical lipoproteins that act as a membrane scaffold, surrounding bilayer fragments in which the membrane protein of interest is embedded [15,16,17].

In contrast to the use of amphipols, bicelles and nanodiscs, which require extraction of proteins from membranes with potentially destabilising detergents and their subsequent reconstitution into a non-natural lipid environment, the use of styrene maleic acid co-polymer (SMA) enables detergent-free isolation of membrane proteins and retention, at least to some extent, of their native lipid environment. The SMA scaffold has been used for many years in the plastics and pharmaceutical industries and has recently been applied to investigation of membrane proteins [18,19]. The alternating hydrophobic (styrene) and hydrophilic (maleic acid) moieties of SMA render it amphipathic and capable of inserting into biological membranes. This results in the extraction of small discs of lipid bilayer, typically containing an integral membrane protein, encircled by polymer and termed SMA lipid particles (SMALPs). Such SMALPs are water-soluble and if the encapsulated protein is suitably tagged they can be purified by standard affinity chromatographic methods. Importantly, this detergent-free method of membrane protein purification, with partial retention of the natural lipid environment, results in maintenance of protein function, as recently demonstrated for the bacterial outer membrane enzyme PagP and for several eukaryotic members of the ABC transporter family [18,19,20].

The development of direct detectors and more stable microscopes, combined with new data processing algorithms and improved specimen preparations, has recently resulted in determination at atomic resolution of several protein structures, including a eukaryotic ion channel, by electron microscopy (EM), using approaches not reliant on symmetry or 2D crystallisation [21,22,23]. Although X-ray crystallography still typically allows higher resolutions, obtaining highly ordered crystals can make structure determination problematic. In contrast, single particle electron microscopy circumvents the need for crystals and although small proteins can be difficult to visualise, due to the poor signal to noise ratio, it is possible to obtain high resolution structural information on membrane proteins by this route. Here we report the use of an SMA co-polymer for extracting and purifying a membrane protein, facilitating its structural investigation by negative stain EM. The *Escherichia coli* multidrug transporter AcrB [24], which in its native trimeric state has an approximate size of ~ 360 kDa, was used as a model system, representative of many transport proteins. We show that the ease of membrane protein extraction in an SMA scaffold, combined with negative stain EM, provides an efficient and rapid approach for studying the structures of membrane proteins in their native lipid environments.

## 2 Materials and methods

### 2.1 Protein overexpression and purification

Mixed inner and outer *Escherichia coli* membranes harbouring AcrB, bearing a C-terminal octahistidine tag, were prepared as described previously [25]. For preparation of SMALPs, membranes (45 mg) were incubated with gentle shaking for 2 h at room temperature in 32 mL 50 mM Tris–HCl, pH 8.0, containing 500 mM NaCl, 10% (w/v) glycerol and 2.5% (w/v) SMA (SMA polymer was synthesised as previously described [19]). The insoluble fraction was removed by centrifugation at 4 °C for 1 h at 100,000 *g*_av_ and then the supernatant was incubated overnight at 4 °C with 2 mL of HisPur™ Cobalt Resin (Thermo scientific) with gentle agitation. The resin was subsequently washed with 10 column volumes of 50 mM Tris–HCl, pH 8.0, containing 500 mM NaCl and 10% (v/v) glycerol before elution of the AcrB-containing SMALPs with the same buffer containing 300 mM imidazole. Before subsequent analyses, imidazole was removed by dialysis against the same buffer without imidazole. Preparations containing high or low salt concentrations were prepared by dialysis against 50 mM Tris–HCl, pH 8.0, containing 5% (v/v) glycerol and 1 M or 10 mM NaCl, respectively.

### 2.2 Electron microscopy

Negative stain grids were prepared by applying 3 μL of protein solution (~ 20 μg/mL) onto a carbon-coated copper grid that had been previously irradiated under a UV lamp for 40 min. The grid was then stained with 1% uranyl acetate. Grids were imaged using a Tecnai T12 microscope fitted with a Tungsten filament operating at 120 kV. A total of 307 micrographs were recorded at a nominal magnification of 30,000× on a 2 k × 2 K Gatan CCD camera resulting in an Å/pixel value of 4.0. The Boxer programme in EMAN 2 was used to hand pick 9346 particles [26]. Reference free 2D classification was carried out in both IMAGIC-5 and Relion 1.3, with the resulting classes being indistinguishable in both methods [27,28]. Those particles which aligned and classified poorly or showed clear “doublet” particles, corresponding to dimers of the homotrimeric AcrB protein, were removed, resulting in 6884 “singlet” particles, corresponding to AcrB homotrimers, which were used in subsequent 3D refinement. Because doublet particles represented a fairly small fraction of the dataset, further micrographs were collected from which doublets were picked and merged to the previous stack. In total 2116 particles were collected of representative doublet particles and these were aligned and classified in the same manner as for the singlets. More diluted grids of SMALPs harbouring AcrB we prepared by applying 10 μg/mL protein and staining as previously described. In total 116 micrographs were collected, resulting in 556 particles. Grids were also prepared as previously described using 20 μg/mL AcrB and 10 mM or 1 M NaCl, with 70 micrographs collected and processed for each sample.

3D reconstructions were generated using the Relion 1.3 3D Classification procedure with a low resolution ellipsoid as a starting model for refinement using C3 symmetry. The reconstruction obtained through this procedure was then low pass filtered to 60 Å and used as a starting model for 3D auto-refinement within Relion resulting in a ~ 27 Å reconstruction, as determined by the gold standard CTF [27]. The auto-refinement procedure was then repeated using the same starting model and C1 symmetry, to check for symmetry artefacts. The resulting reconstruction in C1 was indistinguishable from the model produced via refinement with C3 symmetry imposed. The processing procedures were then repeated with full CTF correction within Relion, leading to an improved resolution of 23 Å in the final model. The model has been deposited within the Electron Microscopy Database (EMDB) with accession number 2714.

### 2.3 Phospholipid assay

The phospholipid assay was carried out by adding 0.45 mL of 8.9 N H_2_SO_4_ to the samples before boiling at 220 °C for 30 min. After cooling, 0.15 mL of H_2_O_2_ was added to the samples before heating for a further 30 min. After addition of 4.9 mL of buffer (0.255% ammonium molybdate (VI), 1% ascorbic acid), the glass tubes were sealed with a screw top lid and placed at 100 °C for 10 min. The absorbances of the resulting solutions were measured at 820 nm and a phosphorus standard was used as a reference (Sigma-Aldrich).

### 2.4 Analytical ultracentrifugation

A Beckman XL-1 analytical ultracentrifuge (Beckman Coulter, Palo Alto, CA, USA) with an eight-cell 50 Ti rotor was used for sedimentation velocity analysis. Purified AcrB–SMALP complex in the presence of high (500 mM) and low (10 mM) NaCl concentrations was prepared using the previously described protocol [20] Two protein samples were loaded into double-sector cells at 0.5 mg mL^− 1^ in either 10 mM NaCl 50 mM Tris pH 8 or 500 mM NaCl 50 mM Tris pH 8. The samples were centrifuged at 40,000 rpm for 20 h at 4 °C and detected at 280 nm. The continuous c(s) analysis method (using a frictional coefficient of 1.9) was used to determine sedimentation coefficients and molecular masses using the SEDFIT software [29].

### 2.5 Fluorescence polarization assay

The rhodamine 6G (R6G) binding affinity of AcrB in SMALP was determined by fluorescence polarization as described [30,31]. Briefly, the AcrB SMALP low salt concentration solutions were titrated into the ligand binding solution (50 mM Tris (pH 8), 10 mM NaCl, 5% glycerol and 1 μM R6G). The fluorescence polarization measurement was taken after incubating the sample for 10 min at 25 °C to ensure that the binding has reached equilibrium. The readings were performed using a BMG LABTECH POLARstar Galaxy plate reader. The excitation and emission filters were 520 and 575 nm, respectively. Titration experiments were repeated four times to achieve an accurate Kd value. The non-linear curve fitting was performed by using the Kaleidagraph programme.

## 3 Results

### 3.1 Preparation and characterisation of AcrB SMALPs

**Fig. 1:**
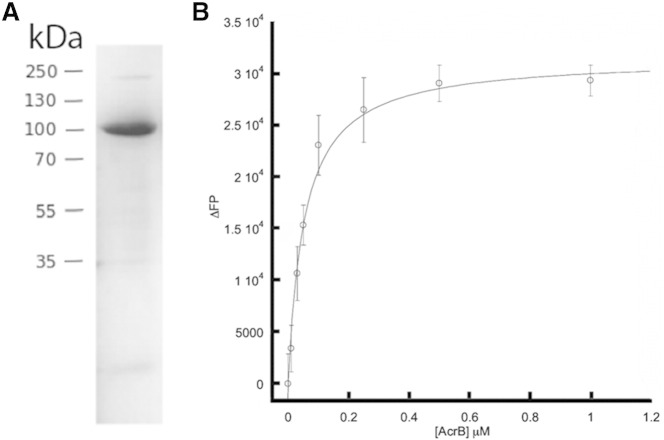
(A) Biochemical characterisation of purified AcrB SMALPs. Coomassie blue-stained SDS-polyacrylamide gel of purified SMALPs. The mobilities of marker proteins of known molecular mass are shown on the left. (B) Representative fluorescence polarization of AcrB SMALP with R6G. Binding isotherm of AcrB SMALP with R6G, shows a KD of 52 ± 6.6 nM, in buffer containing 50 mM Tris (pH 8) 10 mM NaCl and 5% glycerol.

AcrB is a trimeric secondary transporter found in the inner membrane of *Escherichia coli*, which in complex with the outer-membrane channel TolC and the periplasmic protein AcrA is responsible for resistance to dyes such as acridine, to detergents and to many lipophilic antibiotics [32]. Its structure has been solved by X-ray crystallography [24], rendering it a suitable model system to test the use of a combination of SMA-mediated purification and EM analysis to investigate membrane protein structure. Membranes from bacteria expressing AcrB bearing a C-terminal His_8_-tag were incubated with SMA, as described above, to generate SMALPs, which were purified by chromatography on cobalt resin. This procedure yielded a preparation which exhibited essentially a single band of the expected size of 110 kDa when analysed by SDS-PAGE (Fig. 1A).

In order to investigate if the SMALP scaffold maintained AcrB in an active state, fluorescence polarization studies were conducted to measure the Kd value of rhodamine 6G binding. Binding was measured at 52 ± 6.6 nM (Fig. 1B).

### 3.2 Electron microscopy

**Fig. 2:**
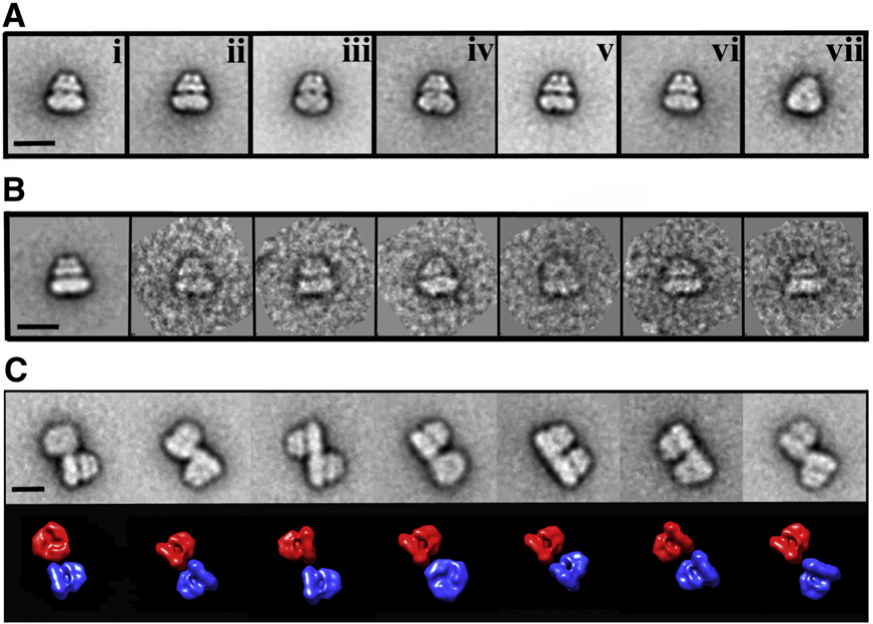
(A) Representative negative stain AcrB classes. The classes are predominantly of a “side” view, equivalent to that viewed from the plane of the bilayer, with an example of a “base” view from the cytoplasmic surface of the protein shown in the far right panel (vii). B) Representative AcrB singlet classum on the left, with some of the raw particles which make up the class shown. C) Classes of the AcrB trimer doublets (top panel), with the orientations shown below (bottom panel) using the AcrB reconstruction. Scale bar represents 15 nm.

Negatively stained grids bearing samples of AcrB SMALPs showed well dispersed protein, comprising a mixture of approximately 70% discrete “singlet” particles and 30% “doublet” particles of twice the size, apparently resulting from association of the singlet particles in pairs. There was no significant aggregation to particles of larger size. The resulting singlet AcrB classes showed clear and distinct features consistent with a 3-fold symmetry (Fig. 2, panel vii) and a structure of approximate dimensions 150 Å height and 150 Å width. Comparison of these classes to the crystal structure of AcrB showed the presence of typical features, such as the dome-like structure which protrudes from the periplasmic surface of the membrane, formed by the pore and TolC docking domains [24]. At the widest part of the particles, opposite to the putative extramembranous dome-like structure, the “base” of the AcrB/SMALP complex singlet particles showed an enlargement consistent with an SMA/phospholipid envelope surrounding the membrane-spanning region of the protein trimer (Fig. 2). The doublet particles appeared to represent dimerisation of the AcrB trimer, with the interface involving the region putatively identified as containing the SMA/phospholipid envelope. To investigate this further a total of 2116 doublet particles were removed from the dataset and processed independently. Alignment and classification of these doublets revealed approximately 7 common arrangements, each of which involved interaction between the putative SMA/phospholipid-containing regions, but with no clear preference for any particular configuration (Fig. 2C). In no instances were any doublets seen in which the interface involved the putative periplasmic or cytoplasmic regions of the AcrB trimer, an observation consistent with AcrB being homotrimeric in its natural state [32]. Formation of doublet particles, possibly involving the sharing of polymer molecules between two singlet particles, might have arisen during SMALP preparation or from interactions occurring on the carbon surface of the grids during sample preparation for microscopy. To investigate the latter possibility, further 116 micrographs were collected of a more dilute sample, which resulted in ~ 5 particles/micrograph, as opposed to ~ 35 for the previous data set. Analysis of the resulting data showed that ~ 25% of the AcrB particles were doublets, a percentage similar to that of 30% seen for the more concentrated sample. It is thus unlikely that doublet formation results from crowding during sample preparation for EM. Further analysis of AcrB trimer doublet formation was conducted by collecting data in the presence of high (1 M) and low (10 mM) NaCl concentrations. These results showed a modest difference, with ~ 15% doublets in the 10 mM NaCl sample and ~ 30% doublets in the 1 M NaCl sample, similar to that seen in the 500 mM NaCl sample. These data suggest that exposure to low ionic strength (10 mM NaCl) significantly reduces the extent of doublet formation, but does not completely prevent it. The lack of any preferred doublet interface, as seen in Fig. 2C, suggests that particle association occurs non-specifically via the SMA/phospholipid envelope and does not involve any defined protein–protein interface. Importantly, whatever its origin, doublet formation does not hinder the ability to generate an accurate 3D reconstruction of the protein moiety of the SMALP.

**Fig. 3:**
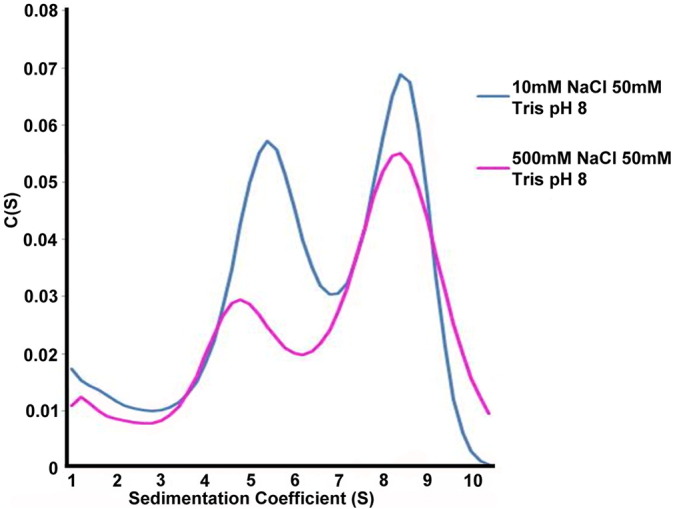
Sedimentation velocity AUC profiles of AcrB SMALP at 10 mM and 500 mM NaCl in 50 mM Tris pH 8. Two distinctive populations are seen, indicating AcrB SMALP as singlet and doublet trimers, with sedimentation coefficients of 4.8 and 8.5 which correspond to Molecular Masses of 405 kDa and 810 kDa of the particles, respectively.

To further investigate the effect of ionic strength on the formation of AcrB–SMALP doublets, AUC was performed in the presence of both 10 mM and 500 mM NaCl. At both salt concentrations two peaks could be clearly identified with sedimentation coefficients (4.8 and 8.5) corresponding to Molecular Masses of 405 kDa and 810 kDa, respectively. In the presence of low NaCl (10 mM), the larger species (810 kDa), which is of equivalent size to a doublet, was reduced by 25% compared to the high salt (500 mM) conditions (Fig. 3). This data was consistent with the EM analysis showing the presence of a 810 kDa species, which is reduced by significantly lowering the salt concentration.

**Fig. 4:**
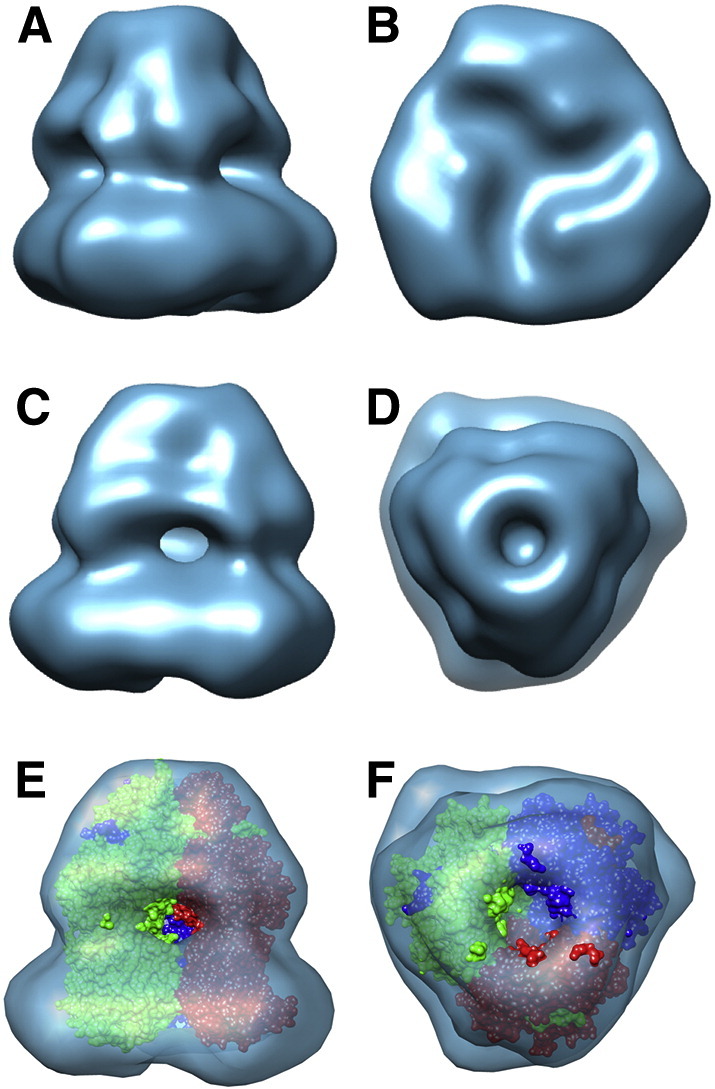
(A) Surface view of the AcrB reconstruction. Clear 3-fold symmetry can be seen from the base ((B) (cytoplasmic face)) and top ((D) (periplasmic face)) of the structure. Fitting of the AcrB crystal structure (PDB ID : 1IWG; [24]) into the reconstruction showing the excellence of fit as seen from the side (E) and top (F). Extra density can be seen surrounding the transmembrane domain of the AcrB structure, which is accounted for by the SMA/phospholipid envelope.

Although EM 2D classes give insightful information on the structure of AcrB, a 3D reconstruction was also generated to obtain further structural detail. The resulting 3D reconstruction, which was generated on a featureless ellipsoid starting model and not the AcrB crystal structure, showed clear similarities to the latter. The 3D reconstruction was generated with either no applied symmetry or with 3-fold symmetry applied to match that of the AcrB crystal structure and consistent with the 3-fold symmetry seen in the classes representing the view of the base (Fig. 4B & D). The AcrB model refined with no symmetry showed clear 3-fold symmetry and the reconstruction was indistinguishable from that processed with 3-fold symmetry. Fitting of the crystal structure within the EM reconstruction showed a very close fit within the extramembranous periplasmic region. The resolution of the structure is such that a “vestibule” area is seen emanating from the core, consistent with the gaps between the individual AcrB monomers. However, there was an excess of density about the membrane spanning region, consistent with the presence of an SMA/phospholipid envelope (Fig. 4B). By calculating the reconstruction volume around this region we can show that ~ 56,500 nm^3^ of this structure is a result of the latter. Interestingly, the SMA/phospholipid envelope follows the contours of the protein structure, more closely resembling a detergent envelope than the larger disk-like structure seen for nanodisc-embedded proteins [33]. The latter can cause problems in image processing due to the dominating effect of the nanodisc in alignment and classification, in comparison with the features of the embedded membrane protein.

To confirm the presence of phospholipids within the SMA scaffold around AcrB, the inorganic phosphate content was measured following acid hydrolysis. This revealed that phospholipids were present at ~ 40 molecules/AcrB trimer. Analysis of the AcrB crystal structure shows that there are 36 transmembrane helices, which is ~ 25% greater than cytochrome oxidase which has 56 +/− 5 motionally restricted lipids associated with it [34]. This suggests ~ 72 lipids might be associated with the surface of the AcrB trimer in the lipid bilayer. Fitting lipids into the EM envelope shows that ~ 80 would be required to fully cover the transmembrane region of AcrB. Moreover, an MD simulation of AcrB insertion into a DPPC bilayer predicts that ~ 103 lipid molecules will be closely associated with the surface of the membrane-spanning portion of the AcrB trimer in the membrane [35]. In all cases these estimates are significantly higher than the 40 molecules calculated through the phosphate assay. This discrepancy suggests that the native lipid alone, encapsulated by the SMA polymer, is insufficient to form a complete annulus around the protein and that the SMA polymer itself, in particular the styrene side chains, intercalates between with the lipid acyl chains and contributes to shielding the hydrophobic surface of the protein from the aqueous environment.

## 4 Conclusions

The use of SMALPs for structure determination by electron microscopy has a number of distinct advantages over conventional detergent-based techniques. The first significant advantage is the relative ease of protein extraction. By removing the need to screen a selection of different, often expensive detergents, the speed and economy of protein extraction can be significantly improved. Moreover, recent developments in electron microscopy have greatly improved the speed of data acquisition, together with the resolution and conformational information obtainable [21,22,36]. Putting these developments together, in the present study we have investigated the use of SMALPs within electron microscopy to study membrane proteins. The negative staining approach employed has the advantage of being relatively quick and easy to perform whilst giving a modest achievable resolution of > 15 Å. For the reconstruction detailed here the negative stain grids were prepared and data collected in one day, with the subsequent particle picking and data processing being carried out in the following week. Structures obtained at such resolutions obtained can provide valuable information on membrane proteins, such as subunit stoichiometry within complexes, and importantly can reveal insights into the conformational changes which accommodate catalytic cycling [37]. The difficulty in obtaining atomic information through X-ray crystallography makes electron microscopy an invaluable technique for understanding protein structure and function. Moreover, the use of antibody labelling and novel tags can reveal information on subunit or inhibitor location [38,39]. The use of single particle cryo-EM, although technically more demanding can provide a significant improvement in the resolution obtainable, with 3.4 Å having been achieved for the TRPV1 ion channel [22]. The SMALP scaffold is highly amenable to single particle cryo-EM as it has the advantage of avoiding detergents within solution, which can diminish the quality of data by lowering the contrast between particle and ice [40]. For example, recent studies have used a SMALP scaffold to investigate the structure of PgP using a cryo-EM approach [20]. However, the process of grid optimisation, data collection and processing of cryo-EM data sets lead to a longer experimental time frame than for the negative stain microscopy approach detailed here.

The most significant advantage to use of the SMALP system is the ability to encapsulate a membrane protein in a more “natural” environment than is provided by detergents. The latter typically possess shorter hydrophobic segments than natural phospholipids and so may perturb both membrane protein structure and function. The combination of a phospholipid analysis and negative stain microscopy has revealed that although a large annulus can be seen surrounding the membrane embedded region of AcrB a significant proportion of this is the SMA polymer itself, with the styrene moieties of the polymer probably intercalating between the acyl chains to complete the hydrophobic environment protecting the membrane-spanning part of the protein. This feature may explain why a significant population of AcrB/SMALP complexes can be seen to form doublets in the EM and AUC experiments. The extent of this doublet formation can be reduced by lowering the ionic strength of the buffer but cannot be completely prevented. This is not a significant problem within EM experiments as doublets can be easily removed by standard data processing techniques but it must be a consideration for other experimental averaging approaches such as, for example, X-ray or neutron scattering. Although the mechanism by which the addition of NaCl increases doublet formation is unknown, it is likely that electrostatic repulsion between the maleic acid moieties of the SMALPS is decreased at high ionic strengths. Studies on the eukaryotic ABC PgP transporter family, showed that activity was maintained in the SMALP scaffold [20]. In order to expand this observation and confirm that AcrB is also active after encapsulation by the SMALP scaffold we conducted a fluorescence polarization assay (Fig. 1B). It is interesting to note that not only did the SMALP scaffold maintain activity for AcrB (52 nM) but this is significantly higher than that reported for the DDM isolated form (5.5 μM) using the same assay conditions [41]. In summary, we have reported how the use of the SMALP system enables rapid structure characterisation using negative stain microscopy, thus providing an important new tool for membrane protein structure determination with the scope for application in a wide range of disciplines.
